# Complete False Anchylosis of Hip Joint—Cure

**Published:** 1872-05

**Authors:** Robert Battey


### COMPLETE FALSE ANCHYLOSIS OF HIP JOINT—CURE.

BY ROBERT BATTEY, M. D., READ BEFORE THE GEORGIA MEDICAL ASSOCIATION.

Mr. George W. K., aged twenty-one, stout and hearty, was attacked with acute rheumatism in the left hip joint about the 10th of December, 1865, for which he was treated by my friend Dr. Gregory. On the 30th of December he came for a time under my care, and, being still unrelieved, he passed, 22d January, into the hands of a "root doctor." An abscess of moderate size had formed near the joint, and was opened by me on the 12th of January.

*May* 24, 1866.—George again applied to me for relief. The joint is completely anchylosed, he is not able to move it a particle, nor am I able to detect a particle of movement under my manipulations. There has been no pain about the joint for four weeks past, all evidences of inflammation are gone, no swelling, no tenderness elicited on pressure about the joint, or in my efforts to move it. The thigh is flexed upon the pelvis to near a right angle with the trunk so that the heel is elevated nearly to a level with the opposite knee, and the whole limb is strongly abducted, in a most unseemly manner, and is greatly in the way of his crutch in walking.

Notwithstanding the diseased action in the joint is gone, and he no longer suffers pain, the awkward position of the anchylosed limb is a serious deformity—it obstructs the free movements of his crutch upon that side; it greatly embarrasses his movements in bed; it necesitates a special adaptation of his nether garments that he may easily put them on and off; but above and beyond all these inconveniences, he is poor, and dependent upon farm labor for subsistence, for which he is incapacitated. It is of the greatest importance to him that he should regain the mobility of the joint, if possible; and if this be not practicable, that the vicious position of the limb should be corrected. It was therefore determined to forcibly break up the adhesions in and around the joint, or fracture the neck of the femur, in the attempt that the limb might at least be brought down more nearly to its natural parallelism to its fellow; and upon this determination he was allowed several weeks upon a course of colchicum and iodide of potassium, to insure a thorough riddance of all remaining rheumatic vice which might endanger the results.

*July* 5.—The patient, having been purged, was submitted to operation, my friend Dr. R. V. Mitchell being present and admintering chloroform. The patient was placed upon a low, firm bed, lying upon the back, the limb was seized with one hand upon the ankle the other upon the knee, and a gradually increasing force was brought to bear, in a series of impulsive jerks, until the full power of my arms was brought into requisition, but without avail. The adhesions were yet firm and unmoved. I now turned the patient upon the right hip, so as to bring his knee to a perpendicular position, and added to my previous efforts with my arms the weight of my own body resting upon his knee, throwing my weight suddenly upon the limb and then withdrawing myself for another similar effort, and so on until one or two loud *snaps* were heard, which reminded one forcibly of the sound of fracturing bone; so loud, indeed, as to bring in the mother from another room to see what had occurred. The limb was now carried to complete flexion, rotated to a limited degree, and extended somewhat beyond the position of anchylosis, so as to form an angle of about 120° with the trunk. The movement of adduction was still quite limited, but on account of the heat of the weather and the full habit of the patient, it was deemed prudent to be content with the rupture obtained, and rely upon gradual extension of the remaining bands. The limb was therefore placed at rest in the position of anchylosis, and a half grain of morphia given.

*July* 6.—Has rested well; no fever; no pain; the joint is very sensitive to every movement.

*July* 8.—Very comfortable every way; talks about sitting up; moved the limb gently for a limited area in every direction; complained of much soreness in these movements, but no very great amount of pain.

*July* 10.—All goes well; had a little diarrhœa yesterday, thinks it was from drinking very freely of chicken soup. No movement to-day; there is neither pain nor tenderness about the joint, except when moved; does not complain when the joint is moved cautiously, and not too far from its position at rest; submitted it to a free passive movement in every direction. The area of movement is considerably enlarged; the knee can now be carried up to the trunk, and can be brought down to form an angle of 160° with the body; adduction is still limited. Directed him to move the joint himself daily, and gradually extend the range of movement as he could bear it.

*July* 14.—Is going on well; has considerable voluntary motion in the joint without pain; extreme movements are still very painful. Allowed to sit up now and resume the use of his crutches and continue the daily movement of the joint.

*July* 18.—Sits up much of the time; no pain; latitude of the motion increasing.

*July* 24.—Complains of pain and swelling in left knee; came on last night after putting his feet into cold water. Directed stimulating liniment and flannel bandage for the knee, and to resume the colchicum and iodide potassium.

*July* 26.—The pain and swelling in the knee are gone; the hip is comfortable; gets about well on his crutches; will continue to exercise the joint.

*November* 1.—He has gone on very well for the past three months without much attention from me. His general health is perfect. There is neither pain nor soreness in the limb or joints. The motion in the hip, although tolerably free in flexion and extension, does not admit of much adduction of the thigh, and seems still tied down by bands of adhesion on the outer aspect of the joint. My friend Dr. Hoyt administering chloroform, I proceeded to break down these bands of adhesion by impulsive force, as in the first instance. This accomplished, the limb moved freely in all directions. I now discovered in the joint movements distinct evidences of two rough sufaces within, rubbing upon each other, a sort of sub-crepitation, which was evident to my sense of touch, and which I seemed to hear as well.

*November* 3.—Doing well, has had no constitutional disturbance, and little or no pain when he lies quiet. Instituted gentle passive movements, which were continued daily with more and more freedom until

*November* 8.—Gave a little chloroform, and moved the joint freely in every direction.

*November* 15.—I have exercised the joint daily by passive movements. He suffers some pain for an hour or two after my manipulations, but rests, on the whole, very comfortably. He is daily gaining strength in the limb, and gets about on the farm very well with his crutches.

*November* 24.—I have exercised the joint every third day since last entry.

*November* 30.—Complains of rheumatic pains in the joint at night, for which he resumes the colchicum and iodide of potassium mixture, which speedily relieved them.

*May* 9, 1867.—Heard from him on a farm in Alabama. He has thrown away his crutches, and is plowing regularly every day in the field. The restoration of the limb, both in form and in function, is as perfect as could be desired.

